# Prognostic significance of resting cardiac power to left ventricular mass and E/e’ ratio in heart failure with preserved ejection fraction

**DOI:** 10.3389/fcvm.2022.961837

**Published:** 2022-08-18

**Authors:** Cong Chen, Jie Zhao, Ruicong Xue, Xiao Liu, Wengen Zhu, Min Ye

**Affiliations:** ^1^Department of Cardiology, The University of Hong Kong-Shenzhen Hospital, Shenzhen, China; ^2^Department of Cardiovascular Medicine, Wuhan Third Hospital, Tongren Hospital of Wuhan University, Wuhan, China; ^3^Department of Cardiology, The First Affiliated Hospital, Sun Yat-sen University, Guangzhou, China; ^4^Department of Cardiology, Sun Yat-sen Memorial Hospital, Sun Yat-sen University, Guangzhou, China

**Keywords:** power/mass, E/e’ ratio, echocardiography, heart failure with preserved ejection fraction, outcomes

## Abstract

**Background:**

Cardiac power-to-left ventricular mass (power/mass) is an index reflecting the muscular hydraulic pump capability of the heart, and the E/e’ ratio is a specific indicator for identifying increased left ventricular filling pressure. Limited data exist regarding the prognostic value of incorporating power/mass and E/e’ ratio in heart failure with preserved ejection fraction (HFpEF).

**Materials and methods:**

In total, 475 patients with HFpEF from the Treatment of Preserved Cardiac Function Heart Failure with an Aldosterone Antagonist (TOPCAT) trial with complete baseline echocardiography data were included in our analysis. Patients were categorized into four groups according to power/mass and E/e’ ratio. The risk of outcomes was examined using Cox proportional hazards models and competing risk models.

**Results:**

Patients with low power/mass and high E/e’ were more likely to be males (60.5%), with higher waist circumference, and had a higher prevalence of diabetes (52.1%), atrial fibrillation (50.4%), and lower estimated glomerular filtration rate (eGFR). Combined resting power/mass and E/e’ have graded correlations with left ventricular (LV) dysfunction and clinical outcomes in patients with HFpEF. After multivariable adjustments, an integrative approach combining power/mass and E/e’ remained to be a powerful prognostic predictor, with the highest HRs of clinical outcomes observed in patients with low power/mass and high E/e’ (all-cause mortality: HR 3.45; 95% CI: 1.69–7.05; *P* = 0.001; hospitalization for heart failure: HR 3.27; 95% CI: 1.60–6.67; *P* = 0.001; and primary endpoint: HR 3.07; 95% CI: 1.73–5.42; *P* < 0.001).

**Conclusion:**

In patients with HFpEF, an echo-derived integrated approach incorporating resting power/mass and E/e’ ratio remained to be a powerful prognosis predictor and may be useful to risk-stratify patients with this heterogeneous syndrome.

**Clinical Trial Registration:**

[https://clinicaltrials.gov], identifier [NCT00094302].

## Introduction

Heart failure with preserved ejection fraction (HFpEF) accounts for approximately half of the patients with heart failure (HF) in the community and leads to substantial morbidity and mortality (1). Although left ventricular ejection fraction (LVEF) is the most frequently used index of cardiac function, it does not accurately reflect myocardial contractility and stratify prognosis well in the HFpEF population, with similar death risk at different values above 50% (2). Cardiac power (CP), which is characterized as a product of cardiac output and mean arterial blood pressure (MBP), reflects the muscular hydraulic pump capability of the heart to maintain circulation. As a comprehensive indicator of cardiac pump function, CP at rest and peak stress has been previously demonstrated to be significantly associated with outcomes in HF (3, 4). In light of more recent conceptual and experimental advances in HF research, a novel concept of CP-to-left ventricular (LV) mass (CP normalized by LV mass), integrating information of ventricular remodeling with cardiac pump function, is instrumental in the stratification of patients with chronic and advanced HF (5–7). Notwithstanding power/mass is an integrated measure reflecting cardiac hydraulic pumping capacity, it did not encompass information about LV diastolic function.

In HFpEF, a normal LVEF may be associated with reduced cardiac output and/or increasing LV filling pressure, which is the intrinsic characteristic of this heterogeneous syndrome. Therefore, a combined assessment of both systolic and diastolic LV functions might be a better approach to decipher pathophysiological mechanisms and determine the prognosis of HFpEF, beyond the simplistic evaluation by LVEF. In recent years, novel hemodynamic classifications taking into account both LV anterograde flow and filling pressure have been proposed in the general HF population and patients with heart failure with reduced EF (HFrEF) (8, 9). However, there are few studies regarding the prognostic impact of incorporating LV pump function and filling pressure in HFpEF. Therefore, our study aimed to assess the joint value of echo-derived resting CP-to-LV mass and E/e’ ratio for risk stratification and prognosis prediction in HF with preserved ejection fraction.

## Materials and methods

### Study population

We used data from the Treatment of Preserved Cardiac Function Heart Failure with an Aldosterone Antagonist (TOPCAT) trial obtained from the National Heart, Lung, and Blood Institute (NHLBI). The principles and procedures of the TOPCAT trial have previously been described (10). In brief, TOPCAT is a prospective, multicenter, randomized, double-blind, placebo-controlled trial, aiming to explore the efficacy of the aldosterone antagonist spironolactone to reduce cardiovascular outcomes in symptomatic HFpEF. A total of 3,445 patients aged older than 50 years with at least one sign and one symptom of HF and a LVEF ≥ 45% were enrolled. Additional eligibility criteria included either elevated natriuretic peptide level within the previous 60 days or hospitalization for HF within the previous 12 months before randomization. Exclusion criteria are severe systemic illness with a life expectancy of <3 years; severe renal dysfunction; severe chronic pulmonary disease; known infiltrative or hypertrophic obstructive cardiomyopathy; known pericardial constriction; heart transplant; or known chronic hepatic disease.

In this study, we performed a retrospective analysis in the TOPCAT trial and further excluded patients with inadequate information regarding baseline echocardiographic measures including E/e’ ratio (septal) and the variables required for calculating power/mass, as well as potential clinical confounders [including New York Heart Association (NYHA) functional class, medical history, and heart rate]. Finally, a total of 475 patients were included in our analysis. This study was approved by the Medical Ethical Committee of The First Affiliated Hospital, Sun Yat-sen University and conformed with the principles outlined in the Declaration of Helsinki.

### Echocardiographic methods

In the TOPCAT trial, standard measurements of the echocardiographic study were performed according to the recommendations of the American Society of Echocardiography and analyzed at a dedicated core laboratory blinded to randomized treatment assignment and clinical information, as previously described (11, 12). The details of the design and baseline findings of the TOPCAT echocardiographic sub-study, including reproducibility indicator for conventional echocardiographic measures, have been previously published (13).

Cardiac power-to-LV mass (in W/100 g) was calculated using the following equation: Power/mass = mean arterial pressure (mmHg) × stroke volume (L) × heart rate (bpm) × K/LV mass, where *K* = 0.222 (a conversion constant to W/100 g of LV myocardium). Mean arterial pressure (mmHg) was calculated as: Mean BP = [(systolic blood pressure−diastolic blood pressure)/3] + diastolic blood pressure. According to the median of power/mass (0.362 W/100 g) and a septal E/e’ ratio threshold of 15 at baseline, patients were categorized into four groups:

High cardiac power with non-increased filling pressure (**HP-NF**): power/mass > median (0.362 W/100 g) and E/e’ ratio ≤ 15,

Low cardiac power with non-increased filling pressure (**LP-NF**): power/mass ≤ 0.362 W/100 g and E/e’ ratio ≤ 15,

High cardiac power with increased filling pressure (**HP-HF**): power/mass > 0.362 W/100 g and E/e’ ratio > 15,

Low cardiac power with increased filling pressure (**LP-HF**): power/mass ≤ 0.362 W/100 g and E/e’ ratio > 15.

### Outcomes

The primary outcomes of this study were all-cause mortality, hospitalization for HF, and primary endpoints. In the TOPCAT trials, the primary endpoint is a composite of cardiovascular mortality, aborted cardiac arrest, or hospitalization for the management of HF. To analyze death in detail, cardiovascular and non-cardiovascular death were assessed as secondary outcomes in this study. The detailed definitions of these outcomes have been previously described (10). All study outcomes were adjudicated by a clinical endpoint committee according to pre-specified criteria.

### Statistical analysis

Continuous variables were expressed either as mean ± SD or as median (interquartile range) according to the distribution. Categorical variables were presented as numbers with proportions (%). Baseline characteristics and echocardiographic parameters among the pre-specified four groups were compared using ANOVA or Kruskal–Wallis test for continuous variables and using chi-square tests or Fisher’s test for categorized variables as appropriate. Kaplan–Meier curves were constructed to assess the unadjusted cumulative incidence estimates of each outcome stratified by median power/mass and an E/e’ threshold of 15 for the cohort and compared using log-rank tests or gray’s test as appropriate. The prognostic relevance of combined power/mass and E/e’ ratio for all-cause mortality, hospitalization for HF, and primary endpoints were assessed using crude and multivariable-adjusted Cox regression models (reference group: high power/mass with low E/e’ ratio). The risk of cardiovascular and non-cardiovascular death associated with each group was assessed using univariable and multivariable competing risk regression models. Model 1 was adjusted for age, sex, race, region of enrollment (Americas vs. Russia/Georgia), and randomization group. Model 2 was additionally adjusted for NYHA functional class, stroke, atrial fibrillation, heart rate, creatinine, and LVEF. The associations of power/mass or E/e’ ratio with outcomes were also analyzed by crude and multivariate regression analysis with adjustment for the same aforementioned covariates. All the statistical analyses were performed using the R statistical software (R version 4.0.3), and statistical significance was defined as a two-tailed *P*-value of <0.05.

## Results

Among the 475 patients with HFpEF (mean age: 70.6 ± 9.6 years; 48.6% men) included in this analysis, the median of power/mass was 0.362 W/100 g, and 48% had a septal E/e’ ratio higher than 15. There were 129 (27.2%) patients with high power/mass with low E/e’, 118 (24.8%) with low power/mass with low E/e’, 109 (22.9%) with high power/mass with high E/e’, and 119 (25.1%) with low power/mass with high E/e’.

The baseline clinical characteristics according to the pre-specified four groups are presented in Table 1. Patients with low power/mass and high E/e’ were more frequently males (60.5%), with larger waist circumference (109.25 ± 17.96 cm), and had a higher prevalence of diabetes (52.1%) and atrial fibrillation (50.4%). There were also noticeable variations in brain natriuretic peptide (BNP) value and estimated glomerular filtration rate (eGFR) among the four groups, with the highest BNP (median: 300.50 pg/ml) and lowest eGFR (median: 57.35 ml/min × 1.73 m^2^) in patients with low power/mass and high E/e’. As for medication prescription, diuretics were more frequently prescribed (92.4%) in the LP-HF group.

**TABLE 1:** Baseline characteristics of HFpEF patients by power/mass and E/e’ ratio categories.

Characteristic	Overall (*n* = 475)	High power/mass with low E/e’ (*n* = 129)	Low power/mass with low E/e’ (*n* = 118)	High power/mass with high E/e’ (*n* = 109)	Low power/mass with high E/e’ (*n* = 119)	*P*-value
Randomization to spironolactone, *n* (%)	243 (51.2)	59 (45.7)	63 (53.4)	53 (48.6)	68 (57.1)	0.291
Age, years	70.6 ± 9.6	68.0 ± 8.8	72.1 ± 9.3	68.4 ± 9.8	73.9 ± 9.4	<0.001
Male, *n* (%)	231 (48.6)	61 (47.3)	58 (49.2)	40 (36.7)	72 (60.5)	0.005
BMI, kg/m^2^	32.83 ± 7.14	32.43 ± 7.07	33.04 ± 7.30	32.72 ± 6.91	33.16 ± 7.33	0.852
Waist circumference, cm	106.31 ± 16.28	104.80 ± 15.89	107.84 ± 15.98	102.78 ± 14.21	109.25 ± 17.96	0.021
Height, cm	166.58 ± 10.62	167.33 ± 10.53	167.11 ± 10.65	164.49 ± 9.69	167.14 ± 11.35	0.138
Race category, *n* (%)						0.237
White	397 (83.6)	114 (88.4)	99 (83.9)	83 (76.1)	101 (84.9)	
Black	67 (14.1)	13 (10.1)	17 (14.4)	23 (21.1)	14 (11.8)	
All others	11 (2.3)	2 (1.6)	2 (1.7)	3 (2.8)	4 (3.4)	
Heart rate, bpm	68.37 ± 10.92	71.60 ± 10.15	64.87 ± 9.21	71.66 ± 11.36	65.33 ± 11.03	<0.001
SBP, mmHg	126.76 ± 15.61	128.28 ± 13.30	124.34 ± 16.03	130.58 ± 14.32	124.03 ± 17.77	0.002
DBP, mmHg	72.08 ± 10.53	76.35 ± 8.71	71.07 ± 10.05	73.62 ± 10.22	67.03 ± 10.87	<0.001
**NYHA functional class, *n* (%)**						
I–II	293 (61.7)	84 (65.1)	74 (62.7)	59 (54.1)	76 (63.9)	0.312
III–IV	182 (38.3)	45 (34.9)	44 (37.3)	50 (45.9)	43 (36.1)	
Current smoker, *n* (%)	36 (7.6)	13 (10.1)	8 (6.8)	11 (10.1)	4 (3.4)	0.154
Ever smoking, *n* (%)	210 (47.8)	45 (38.8)	59 (53.6)	41 (41.8)	65 (56.5)	0.017
Alcohol, drinks/week						0.878
0	357 (75.2)	92 (71.3)	90 (76.3)	85 (78.0)	90 (75.6)	
1–5	89 (18.7)	29 (22.5)	21 (17.8)	16 (14.7)	23 (19.3)	
5–10	21 (4.4)	5 (3.9)	5 (4.2)	7 (6.4)	4 (3.4)	
11+	8 (1.7)	3 (2.3)	2 (1.7)	1 (0.9)	2 (1.7)	
HF hospitalization, *n* (%)	304 (64.0)	92 (71.3)	71 (60.2)	61 (56.0)	80 (67.2)	0.062
Elevated BNP level, *n* (%)	277 (58.3)	55 (42.6)	71 (60.2)	72 (66.1)	79 (66.4)	<0.001
**Co-morbidities, *n* (%)**						
Previous HF	306 (64.4)	90 (69.8)	75 (63.6)	64 (58.7)	77 (64.7)	0.362
Previous MI	129 (27.2)	32 (24.8)	29 (24.6)	26 (23.9)	42 (35.3)	0.148
PCI	103 (21.7)	23 (17.8)	20 (16.9)	29 (26.6)	31 (26.1)	0.134
CABG	69 (14.5)	15 (11.6)	19 (16.1)	11 (10.1)	24 (20.2)	0.117
Ischemic heart failure	192 (40.4)	49 (38.0)	45 (38.1)	42 (38.5)	56 (47.1)	0.405
Peripheral arterial disease	48 (10.1)	7 (5.4)	13 (11.0)	14 (12.8)	14 (11.8)	0.214
Diabetes mellitus	197 (41.5)	42 (32.6)	37 (31.4)	56 (51.4)	62 (52.1)	<0.001
Hypertension	441 (92.8)	119 (92.2)	107 (90.7)	102 (93.6)	113 (95.0)	0.617
Stroke	47 (9.9)	13 (10.1)	8 (6.8)	11 (10.1)	15 (12.6)	0.517
Dyslipidemia	351 (73.9)	86 (66.7)	84 (71.2)	87 (79.8)	94 (79.0)	0.057
COPD	68 (14.3)	15 (11.6)	16 (13.6)	16 (14.7)	21 (17.6)	0.593
Atrial fibrillation	195 (41.1)	50 (38.8)	58 (49.2)	27 (24.8)	60 (50.4)	<0.001
QRS duration, ms	94.00 [84.00, 110.00]	90.00 [84.00, 102.00]	92.00 [82.50, 113.25]	92.00 [82.75, 108.50]	100.00 [86.00, 123.00]	0.036
**Laboratory values**						
Creatinine	±	1.04 ± 0.29	1.17 ± 0.34	1.12 ± 0.35	1.24 ± 0.33	<0.001
eGFR, mL/min × 1.73 m^2^	62.81 [51.69, 77.25]	70.51 [59.14, 83.30]	61.34 [50.87, 74.28]	65.76 [50.82, 81.23]	57.35 [47.39, 70.28]	<0.001
K, mmol/L	4.23 ± 0.43	4.24 ± 0.46	4.23 ± 0.44	4.22 ± 0.45	4.21 ± 0.37	0.963
BNP, pg/ml (*n* = 204)	239.50 [136.50, 436.75]	175.00 [108.00, 286.50]	265.00 [146.00, 490.00]	257.50 [134.50, 498.25]	300.50 [177.75, 608.50]	0.004
**Medications, *n* (%)**						
Diuretics	392 (82.5)	94 (72.9)	100 (84.7)	88 (80.7)	110 (92.4)	0.001
Beta blocker	376 (79.2)	102 (79.1)	100 (84.7)	87 (79.8)	87 (73.1)	0.179
Statin	321 (67.6)	74 (57.4)	80 (67.8)	79 (72.5)	88 (73.9)	0.023
ACEI/ARB	380 (80.0)	96 (74.4)	99 (83.9)	85 (78.0)	100 (84.0)	0.163
CCB	203 (42.7)	60 (46.5)	45 (38.1)	46 (42.2)	52 (43.7)	0.609
Warfarin	152 (32.0)	36 (27.9)	44 (37.3)	25 (22.9)	47 (39.5)	0.021
Aspirin	318 (66.9)	90 (69.8)	65 (55.1)	82 (75.2)	81 (68.1)	0.010

Values are presented as mean ± SD, median [IQR] or n (%).

HF hospitalization represents patients enrolled in TOPCAT trial with at least one hospital admission in the last 12 months.

Elevated BNP level represents patients enrolled in TOPCAT trial with brain natriuretic peptide in the last 30 days ≥ 100 pg/ml or N-terminal pro-BNP ≥ 360 pg/ml.

HFpEF, heart failure with preserved ejection fraction; BMI, body mass index; bpm, beat per minute; SBP, systolic blood pressure; DBP, diastolic blood pressure; BNP, brain natriuretic peptide; MI, myocardial infarction; PCI, percutaneous coronary intervention; CABG, coronary artery bypass grafting; COPD, chronic obstructive pulmonary disease; ECG, electrocardiogram; eGFR, estimated glomerular filtration rate; HF, heart failure; LVEF, left ventricular ejection fraction; NYHA, New York Heart Association; ACEI, angiotensin, converting enzyme inhibitor; ARB, angiotensin receptor blocker; CCB, Calcium channel blocker.

During a median follow-up of 3.0 years, 88 (18.5%) deaths occurred, of which 57 (12.0%) were adjudicated as cardiovascular deaths. In total, 87 (18.3%) experienced HF hospitalizations and 127 (26.7%) primary endpoints.

### Echocardiographic measures

The baseline echocardiographic characteristics according to the four groups are shown in Supplementary Table 1. There were considerable variations regarding parameters of LV structure, systolic function, and diastolic function among the four groups [all *P* < 0.05 except for left ventricular end-systolic volume (LVESV)]. Objective evidence of LV diastolic dysfunction (LVDD) was found in 90.5% of patients with HFpEF in this study, of which significant LVDD (classified as moderate to severe) accounted for more than one-half of the patients. Although LVDD existed both in the overall patients of HP-HF group (high power/mass with high E/e’) and LP-HF group (low power/mass with high E/e’), the distribution of LVDD grade was distinct between the two groups. Moderate LVDD was more frequent (accounting for 61.3%) in the HP-HF group, while moderate and severe LVDD were both more common in the LP-HF group (accounting for 45.3 and 46.5%, respectively), which indicated a trend toward the deterioration of LVDD from HP-HF group to LP-HF group. Besides, the Left atrial volume (LAV) value of patients in the LP-HF group was significantly greater than that of the other three groups (*P* < 0.001). There were increasing trends of peak E wave velocity to peak A wave velocity (E/A ratio) and peak tricuspid regurgitation velocity from the HP-NF group to the LP-HF group (*P* < 0.001).

### Outcomes

Values of power/mass in patients suffering from adverse outcomes were lower as compared with those free from the outcomes, although the difference between the survivors and non-survivors for cardiovascular death did not reach statistical significance (Supplementary Figure 1). Patients with low power/mass (≤0.362 W/100 g) experienced a higher risk of HF hospitalization (adjusted hazard ratio 1.74, 95% CI: 1.08–2.8, *P* = 0.023) and primary endpoint (adjusted hazard ratio 1.58, 95% CI: 1.08–2.33, *P* = 0.020) (Supplementary Table 2), whereas the HRs of low resting power/mass with mortality outcomes did not reach statistical significance. In contrast, values of the E/e’ ratio were significantly higher in patients suffering from adverse outcomes than in those free from the outcomes (Supplementary Figure 2). In multivariate regression analysis, a high E/e’ ratio (>15) was significantly associated with a greater risk of poor outcomes in patients with HFpEF (Supplementary Table 3).

**FIGURE 1:**
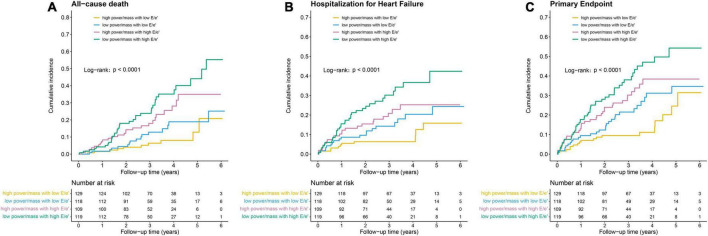
Kaplan–Meier survival curves for **(A)** all-cause death, **(B)** hospitalization for heart failure, and **(C)** primary endpoint.

Outcomes differed by power/mass and E/e’ groups, with a trend toward increased risk of poor outcomes from the HP-NF group to the LP-HF group. The cumulative incidences of primary outcomes according to the pre-specified four groups are shown in Table 2 and Figure 1. In comparison with an incidence rate of 2.4 (95% CI: 1.1–4.3) per 100 person-years in patients with high power/mass and low E/e’, the all-cause mortality incidence rate in those with low power/mass and high E/e’ was 10.9 (95% CI: 7.7–15.0) per 100 person-years. In multivariate analysis, after adjusting for demographic and clinical covariates, patients in the LP-HF group (low power/mass with high E/e’) still had the highest risk of all-cause mortality (adjusted hazard ratio 3.45, 95% CI: 1.69–7.05, *P* = 0.001). Similar association patterns were also observed with HF hospitalization and primary endpoint (Table 2; Figure 1). The incidence rate per 100 person-years of HF hospitalization was highest in the LP-HF group (12.1, 95% CI: 8.4–16.8) and lowest in the HP-NF group (2.7, 95% CI: 1.3–4.8). Patients with low power/mass and high E/e’ experienced more than three times greater risk of HF hospitalization (adjusted hazard ratio 3.27, 95% CI: 1.60–6.67, *P* = 0.001) than those with high power/mass with low E/e’ (Table 3). With regards to the primary endpoint, the incidence rate per 100 person-years (16.2, 95% CI: 11.9–21.6) in the LP-HF group was more than three times higher than that in the HP-NF group (4.4, 95% CI: 2.6–7.0). Patients in the LP-HF group had three times greater adjusted risk of primary endpoint than did patients in the HP-NF group (adjusted hazard ratio 3.07, 95% CI: 1.73–5.42, *P* < 0.001) (Table 3).

**TABLE 2:** Risk of all-cause death, hospitalization for heart failure and primary endpoint in HFpEF patients by power/mass and E/e’ ratio categories.

Outcome	Event rates	Incidence rates, per 100 person-years	Unadjusted		Model 1		Model 2	
			HR (95% CI)	*P*-value	HR (95% CI)	*P*-value	HR (95% CI)	*P*-value
**All-cause death**								
High power/mass with low E/e’	10 (7.8)	2.4 (1.1–4.3)	1.00 (ref)		1.00 (ref)		1.00 (ref)	
Low power/mass with low E/e’	16 (13.6)	4.2 (2.4–6.8)	1.72 (0.78–3.79)	0.180	1.52 (0.68–3.36)	0.304	1.45 (0.65–3.24)	0.361
High power/mass with high E/e’	25 (22.9)	7.7 (5.0–11.3)	3.30 (1.58–6.87)	0.001	3.39 (1.63–7.07)	0.001	3.28 (1.57–6.84)	0.002
Low power/mass with high E/e’	37 (31.1)	10.9 (7.7–15.0)	4.68 (2.32–9.41)	<0.001	3.97 (1.96–8.06)	<0.001	3.45 (1.69–7.05)	0.001
**Hospitalization for heart failure**								
High power/mass with low E/e’	11 (8.5)	2.7 (1.3–4.8)	1.00 (ref)		1.00 (ref)		1.00 (ref)	
Low power/mass with low E/e’	19 (16.1)	5.5 (3.3–8.6)	2.02 (0.96–4.24)	0.064	1.42 (0.67–3.01)	0.360	1.61 (0.75–3.46)	0.223
High power/mass with high E/e’	22 (20.2)	7.6 (4.8–11.5)	2.73 (1.33–5.64)	0.006	2.10 (1.01–4.37)	0.047	1.85 (0.88–3.85)	0.102
Low power/mass with high E/e’	35 (29.4)	12.1 (8.4–16.8)	4.24 (2.15–8.37)	<0.001	2.68 (1.34–5.37)	0.005	3.27 (1.60–6.67)	0.001
**Primary endpoint**								
High power/mass with low E/e’	18 (14.0)	4.4 (2.6–7.0)	1.00 (ref)		1.00 (ref)		1.00 (ref)	
Low power/mass with low E/e’	28 (23.7)	8.1 (5.4–11.7)	1.84 (1.02–3.33)	0.044	1.50 (0.82–2.74)	0.185	1.74 (0.94–3.21)	0.076
High power/mass with high E/e’	34 (31.2)	11.8 (8.2–16.5)	2.64 (1.49–4.67)	0.001	2.38 (1.34–4.25)	0.003	2.18 (1.22–3.89)	0.009
Low power/mass with high E/e’	47 (39.5)	16.2 (11.9–21.6)	3.60 (2.09–6.20)	<0.001	2.75 (1.57–4.82)	<0.001	3.07 (1.73–5.42)	<0.001

Model 1: adjusted for age, sex, race, region of enrollment (Americas vs. Russia/Georgia) and randomization group.

Model 2: adjusted for model 1, additionally adjusted for NYHA functional class, stroke, atrial fibrillation and heart rate, creatinine and LVEF.

**TABLE 3:** Risk of cardiovascular and non-cardiovascular death in HFpEF patients by power/mass and E/e’ ratio categories.

Outcome	Event rates	Incidence rates, per 100 person-years	Unadjusted		Model 1		Model 2	
			HR (95% CI)	*P*-value	HR (95% CI)	*P*-value	HR (95% CI)	*P*-value
**Cardiovascular death**								
High power/mass with low E/e’	8 (6.2)	1.9 (0.8–3.7)	1.00 (ref)		1.00 (ref)		1.00 (ref)	
Low power/mass with low E/e’	11 (9.3)	2.9 (1.4–5.1)	1.47 (0.59–3.63)	0.410	1.33 (0.52–3.41)	0.550	1.32 (0.50–3.51)	0.570
High power/mass with high E/e’	16 (14.7)	4.9 (2.8–8.0)	2.47 (1.07–5.74)	0.035	2.93 (1.21–7.08)	0.017	2.67 (1.09–6.57)	0.032
Low power/mass with high E/e’	22 (18.5)	6.5 (4.1–9.8)	3.23 (1.43–7.26)	0.005	2.71 (1.10–6.71)	0.031	2.43 (0.96–6.13)	0.060
**Non-cardiovascular death**								
High power/mass with low E/e’	2 (1.6)	0.5 (0.1–1.7)	1.00 (ref)		1.00 (ref)		1.00 (ref)	
Low power/mass with low E/e’	5 (4.2)	1.3 (0.4–3.0)	2.64 (0.51–13.66)	0.250	1.96 (0.36–10.70)	0.440	2.36 (0.42–13.29)	0.330
High power/mass with high E/e’	9 (8.3)	2.8 (1.3–5.2)	5.53 (1.19–25.70)	0.029	5.07 (1.05–24.52)	0.043	4.93 (1.05–23.20)	0.044
Low power/mass with high E/e’	15 (12.6)	4.4 (2.5–7.3)	8.42 (1.94–36.56)	0.004	6.08 (1.35–27.43)	0.019	6.64 (1.49–29.60)	0.013

Model 1: adjusted for age, sex, race, region of enrollment (Americas vs. Russia/Georgia) and randomization group.

Model 2: adjusted for model 1, additionally adjusted for NYHA functional class, stroke, atrial fibrillation and heart rate, creatinine and LVEF.

**FIGURE 2:**
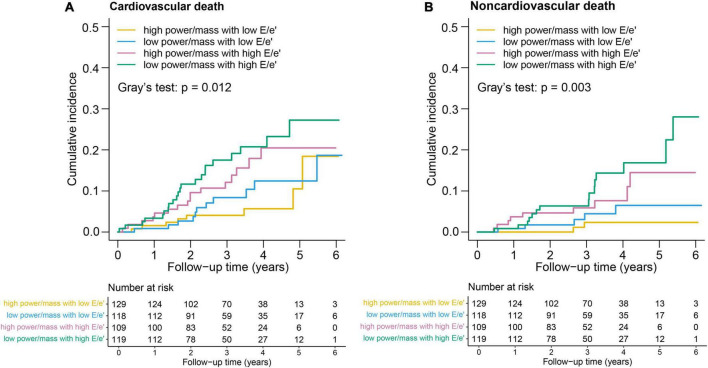
Kaplan–Meier survival curves for **(A)** cardiovascular death and **(B)** noncardiovascular death.

For cardiovascular death, the incidence rate in the LP-HF group (6.5; 95% CI: 4.1–9.8) was more than three times higher than that in the HP-NF group (1.9; 95% CI: 0.8–3.7) (Table 3; Figure 2). Likewise, a significantly higher rate of non-cardiovascular death was also observed in the LP-HF group (4.4; 95% CI: 2.5–7.3), in an unadjusted analysis. After controlling for demographic and clinical covariates, patients in the LP-HF group still had the highest risk of non-cardiovascular death (adjusted hazard ratio 6.64, 95% CI: 1.49–29.6, *P* = 0.013), compared with the HP-NF group (Table 3), whereas HR of cardiovascular death was highest in the HP-HF group (adjusted hazard ratio 2.67, 95% CI: 1.09–6.57, *P* = 0.032) as compared with the HP-NF group.

## Discussion

In this study, we proposed an integrated risk-stratification approach incorporating resting CP-to-LV mass and E/e’ ratio, which allowed effective prognostication in HFpEF. In general, we found that patients with low power/mass and high E/e’ had substantially higher crude rates of poor outcomes than patients with high power/mass and low E/e’. After adjustment for clinical covariates, combining power/mass and E/e’ ratio had a graded relationship with LV dysfunction and clinical outcomes in patients with HFpEF. The predictive value of the integrated approach was stronger than that of the individual variables contained in the approach alone.

An appropriate HF categorization not only should be associated with pathophysiological mechanisms but also have a prognostic significance. A recent study demonstrated that HF phenotypes categorized by LVEF (HFrEF and HFpEF) do not match the pathophysiological perspective of HF (14). Although echocardiographic LVEF is a good outcome predictor in patients with systolic dysfunction (15), it is less useful in evaluating hemodynamics of LV contractility and detecting impaired systolic mechanics in patients with HFpEF (16). Prior studies have evidenced that LVEF alone was not predictive of outcomes in HFpEF (17, 18), which was also confirmed by the echocardiographic sub-study of the TOPCAT trial (11). Cardiac power, which incorporates both the antegrade flow and pressure-generating capability of the heart, shed new light on assessing cardiac pumping function and stratifying patients with HF beyond LVEF. Numerous studies have demonstrated CP to be a robust metric in the evaluation of patients with HF. In particular, peak CP measured non-invasively or invasively has been previously shown to exhibit good performance for prognosis prediction and risk stratification in congestive HF with reduced EF (4, 7, 19). A similar prognostic value was also shown in patients with cardiogenic shock (20), critical cardiac illness (21), and ischemic cardiomyopathy (6). Subsequently, in consideration of the prognostic impact of LV remodeling in HF (13), Dini et al. (5) proposed a novel index “power/mass” (CP output to LV mass), incorporating the information of ventricular geometry, and similar to ejection fraction, which is the ratio between stroke volume and LV end-diastolic volume. It was shown that peak power/mass could effectively distinguish and risk-stratify patients with advanced HF (LVEF ≤ 35%) and those with stable coronary artery disease (22). More recently, Anand et al. (23) evidenced that peak CP-to-LV mass (power/mass) during exercise stress echocardiography was a powerful predictor of prognosis in patients with normal EF. Nevertheless, data regarding the prognostic performance of resting power/mass in HF remained controversial (20, 23–25). A previous study conducted by Cotter et al. (26) has demonstrated that CP index at baseline was a strong prognostic predictor in exacerbated systolic congestive heart failure (CHF). Grodin et al. (24) analyzed 495 patients with advanced HF and found that invasive resting CP index had incremental predictive value for prognosis beyond traditional hemodynamic and cardio-renal risk factors. On the contrary, Anand et al. (23) have shown that baseline resting values of CP was not significantly predictive of mortality and new HF diagnosis at follow-up in patients with normal EF. Following this finding, our results demonstrated that baseline resting power/mass, although statistically different between the survivors and non-survivors, was not as good to predict mortality outcomes in patients with HFpEF. Notably, our analysis also found that baseline resting power/mass was independently associated with a higher risk of HF hospitalization and primary outcomes.

Left ventricular diastolic dysfunction is an important pathophysiological mechanism underlying HFpEF (27) but manifests inadequate sensitivity (28) and specificity (29) in diagnostic and prognostic performance. In recent years, several potential pathobiological mechanisms beyond LVDD have been proposed, such as microvascular function, inflammation, subtle systolic impairment, and inadequate response during exertion (12, 30). A study by Coiro et al. (31) reported that the development of pulmonary congestion during exercise was an independent predictor of adverse outcomes in patients with HFpEF, superior to the performance of the E/e’ ratio. A reduced cardiac output and an elevated LV filling pressure are unifying hemodynamic characteristics of HFpEF, but one does not necessarily predict the other. Therefore, an integrated approach incorporating cardiac antegrade flow and LV filling pressure may be imperative to improve the characterization of pathophysiological mechanisms underlying the functional status and confer complementary prognostic information in HFpEF beyond that provided by single index (14). The ratio of early diastolic mitral inflow velocity to early diastolic mitral annulus velocity (E/e’ ratio) derived from echocardiography is a robust diastolic index that reflects left ventricular filling pressure (LVFP) and is associated with outcomes in HF (32). Despite its modest correlations with outcomes in HFpEF (33), E/e’ ratio displays prominent specificity for identifying patients with increased LV filling pressure (34). A recent study conducted by Dini et al. (35), which involved 727 outpatients with HFrEF, demonstrated that a functional hemodynamic stratification approach based on a cardiac index (using 2.0 L/min/m^2^ as the threshold for low output) and E/e’ ratio (using a value ≥ 15 as a marker of increased LV filling pressure) is useful in predicting survival. A similar pathophysiological stratification approach based on an assessment of cardiac hemodynamics (ventricular forward flow and filling pressure) has also been proposed in patients with cardiogenic shock (36). Our results extend previously published observations and suggest that combining resting CP/mass with E/e’ could effectively predict clinical outcomes in patients with HFpEF. Of note, we observed a continuously increasing trend toward higher event risk across the pre-specified four groups both for all-cause mortality and death from the non-cardiovascular cause, with the highest risk observed in patients with low power/mass and high E/e’, whereas, for cardiovascular death, the highest event risk was found in patients with high power/mass and high E/e’. These findings suggested that the increased risk of total mortality associated with resting power/mass and E/e’ may be mainly interpreted by the non-cardiovascular cause. It is not completely known why the highest event risk for cardiovascular death and non-cardiovascular death occurred in different groups. A possible explanation may be a relatively strengthening HF therapy, with a higher proportion of medication prescriptions found in patients with low power/mass and high E/e’, to some extent modifying the relationship of power/mass and E/e’ with cardiovascular mortality.

## Limitations

There were several limitations to our study. First, the results of this study were based on a relatively small sample of patients with HFpEF, and given the hemodynamic heterogeneity of HFpEF, prospective research with a larger HFpEF cohort was warranted to confirm these findings, as well as determine the value for clinical decision-making and treatment guidance. Second, the application of the E/e’ ratio in estimating LVFP was not suitable in subjects with significant mitral annular calcification (MAC) (37). As the prevalence of MAC was not available in the dataset, we could not exclude those patients with significant MAC in our analysis. Third, due to the pre-specified inclusion and exclusion criteria in the TOPCAT trial, we could not exclude the presence of selection bias in this study, which might limit the generalization of these findings in the community-based cohorts. Finally, we used only septal E/e’ in the integrated approach because septal information was available for most patients.

## Conclusion

An integrated hemodynamic approach that incorporates resting power/mass and E/e’ has graded correlations with LV dysfunction and clinical outcomes in patients with HFpEF. In particular, the prognosis is worst in the presence of low values of resting power/mass and high values of E/e’ ratio in patients with HFpEF. These findings suggest the potential value of an echo-derived integrated approach for risk stratification and prognosis prediction in patients with HFpEF.

## Data availability statement

The original contributions presented in this study are included in the article/Supplementary material, further inquiries can be directed to the corresponding authors.

## Ethics statement

The study was approved by the institutional review board at all participating institutions, and all participants provided written informed consent at enrollment.

## Author contributions

All authors listed have made a substantial, direct, and intellectual contribution to the work, and approved it for publication.
