# Supplementary material for: Prognostic significance of resting cardiac power to left ventricular mass and E/e’ ratio in heart failure with preserved ejection fraction
Source: Front Cardiovasc Med. 2022 Aug 18;9:961837. doi: 10.3389/fcvm.2022.961837 (PMC9433697; doi:10.3389/fcvm.2022.961837)
Supplement: Supplementary file 1 [file Table_2.DOCX]

**Table S1.** Echocardiographic parameters of HFpEF patients by power/mass and E/e’ ratio categories

| **Measures** | **N** | **Overall** | **high power/mass with low E/e'** | **low power/mass with low E/e'** | **high power/mass with high E/e'** | **low power/mass with high E/e'** | ***P*-Value** |
| --- | --- | --- | --- | --- | --- | --- | --- |
|  |  | **(n = 475)** | **(n = 129)** | **(n = 118)** | **(n = 109)** | **(n = 119)** |  |
| ***LV structure*** |  |  |  |  |  |  |  |
| LVEDV, mL | 475 | 99.05±34.82 | 101.14±32.34 | 90.57±35.86 | 107.25±36.45 | 97.69±33.23 | 0.003 |
| LVESV, mL | 475 | 41.10±21.04 | 39.83±17.31 | 39.58±23.03 | 43.73±21.79 | 41.57±21.97 | 0.418 |
| LVEDD, cm | 475 | 4.79±0.58 | 4.62±0.50 | 4.89±0.62 | 4.69±0.55 | 4.95±0.59 | <0.001 |
| LVESD, cm | 475 | 3.33±0.52 | 3.16±0.44 | 3.43±0.55 | 3.25±0.51 | 3.47±0.52 | <0.001 |
| Mean wall thickness, cm | 475 | 1.18±0.20 | 1.08±0.14 | 1.22±0.19 | 1.11±0.17 | 1.29±0.23 | <0.001 |
| LV mass (g) | 475 | 217.81±70.43 | 182.14±48.84 | 237.09±74.40 | 192.69±54.06 | 260.37±70.73 | <0.001 |
| LVMI (g/m^2^) | 475 | 109.22±31.01 | 91.19±19.52 | 117.85±31.99 | 98.84±24.12 | 129.73±30.79 | <0.001 |
| RWT | 475 | 0.49±0.10 | 0.47±0.07 | 0.50±0.09 | 0.47±0.09 | 0.52±0.14 | <0.001 |
| LV geometry | 475 |  |  |  |  |  | <0.001 |
| Normal Geometry | | 59 (12.4) | 28 (21.7) | 11 (9.3) | 13 (11.9) | 7 (5.9) |  |
| Concentric Remodeling | | 176 (37.1) | 70 (54.3) | 30 (25.4) | 53 (48.6) | 23 (19.3) |  |
| Concentric Hypertrophy | | 199 (41.9) | 27 (20.9) | 62 (52.5) | 30 (27.5) | 80 (67.2) |  |
| Eccentric Hypertrophy | | 41 (8.6) | 4 (3.1) | 15 (12.7) | 13 (11.9) | 9 (7.6) |  |
| Power/mass | 475 | 0.38±0.15 | 0.50±0.12 | 0.27±0.06 | 0.49±0.12 | 0.27±0.06 | <0.001 |
| LAV, mL | 461 | 59.75±23.23 | 57.12±21.07 | 56.91±20.69 | 57.19±18.72 | 67.52±29.14 | <0.001 |
| ***LV systolic function*** | |  |  |  |  |  |  |
| LVEF, % | 475 | 59.67±8.20 | 61.29±6.42 | 57.96±8.35 | 60.59±7.50 | 58.77±9.88 | 0.004 |
| LVEF-t, % | 475 | 58.00±6.34 | 59.58±5.67 | 57.06±5.83 | 58.23±6.75 | 56.99±6.82 | 0.003 |
| GLS, % | 323 | -15.68±3.48 | -16.82±2.85 | -15.74±3.76 | -15.60±3.38 | -14.25±3.54 | <0.001 |
| TDI s’ (septal), cm/s | 472 | 5.79±1.75 | 6.15±1.37 | 6.36±2.46 | 5.45±1.18 | 5.11±1.37 | <0.001 |
| ***LV diastolic function*** | |  |  |  |  |  |  |
| E/A ratio | 355 | 1.21±0.63 | 1.04±0.54 | 1.06±0.59 | 1.20±0.53 | 1.55±0.74 | <0.001 |
| TDI e’ (septal), cm/s | 475 | 6.09±2.24 | 6.90±1.92 | 7.28±2.86 | 4.98±1.35 | 5.04±1.39 | <0.001 |
| TDI e’ (lateral), cm/s | 385 | 8.11±3.05 | 8.62±2.90 | 9.09±3.70 | 7.17±2.23 | 7.38±2.78 | <0.001 |
| E/e’ (septal) | 475 | 15.60±6.75 | 10.89±2.73 | 10.52±2.87 | 20.22±5.00 | 21.54±6.20 | <0.001 |
| E/e’ (lateral) | 385 | 11.69±5.81 | 8.83±2.94 | 8.40±2.84 | 14.68±5.61 | 15.83±6.95 | <0.001 |
| Diastolic dysfunction grade (Olmsted) | 348 |  |  |  |  |  | <0.001 |
| Normal |  | 33 (9.5) | 21(22.3) | 12(16.0) | 0 (0) | 0 (0) |  |
| Mild |  | 89 (25.6) | 31 (33.0) | 33 (44.0) | 18 (19.4) | 7 (8.1) |  |
| Moderate |  | 154 (44.2) | 37 (39.4) | 21 (28.0) | 57 (61.3) | 39 (45.3) |  |
| Severe |  | 72 (20.7) | 5 (5.3) | 9 (12.0) | 18 (19.4) | 40 (46.5) |  |
| ***RV structure and function*** | |  |  |  |  |  |  |
| TR jet velocity, cm/s | 290 | 276.17±45.46 | 261.94±34.78 | 268.90±45.53 | 281.24±45.12 | 291.98±49.41 | <0.001 |
| RVFAC, % | 381 | 0.49±0.08 | 0.49±0.06 | 0.49±0.08 | 0.50±0.08 | 0.48±0.08 | 0.314 |
| RVEDA, cm^2^ | 381 | 19.96±6.26 | 18.86±5.81 | 20.34±6.63 | 20.04±5.58 | 20.71±6.86 | 0.179 |
| RVESA, cm^2^ | 381 | 10.20±3.80 | 9.62±3.28 | 10.38±4.27 | 10.02±3.35 | 10.80±4.16 | 0.158 |
| Pulmonary vasular resistance | 191 | 1.90±0.57 | 1.90±0.63 | 1.92±0.53 | 1.79±0.48 | 1.99±0.63 | 0.33 |
| ***Valvular disease*** | |  |  |  |  |  |  |
| Moderate or greater mitral regurgitation | 384 | 41 (10.7) | 7 (7.0) | 12 (13.3) | 9 (10.5) | 13 (12.0) | 0.513 |
| Moderate aortic regurgitation | 474 | 5 (1.1) | 0 (0.0) | 2 (1.7) | 2 (1.9) | 1 (0.8) | 0.467 |
| Significant valvular disease | 465 | 58 (12.5) | 9 (7.1) | 17 (14.7) | 13 (12.5) | 19 (16.0) | 0.164 |

**Abbreviations:* HFpEF, heart failure with preserved ejection fraction; LVEDV, left ventricular end-diastolic volume; LVEDD, left ventricular end-diastolic diameter; LVESV, left ventricular end-systolic volume; LVESD, left ventricular end-systolic diameter; LAV, left atrial volume; LVMI, left ventricular mass indexed for body surface area; RWT, left ventricular relative wall thickness; LVEF: left ventricular ejection fraction by Simpson method; LVEF-t, left ventricular ejection fraction by Teicholtz method; GLS, global longitudinal strain; TDI, tissue Doppler imaging; s’: systolic myocardial velocity; E/A ratio, peak E wave velocity to peak A wave velocity; e’, early diastolic myocardial velocity; E/e’: early diastolic mitral inflow velocity to early diastolic mitral annulus velocity; TR, tricuspid regurgitation; RVFAC, RV fractional area change; RVEDA, RV end diastolic area; RVESA, RV end systolic area.

**Table S2. Risk of clinical outcomes by power/mass median categories**

| **Outcomes** | **Event rate** | **Incidence rates, per 100 person-years** | **Unadjusted** | | **Model 1** | | **Model 2** | |
| --- | --- | --- | --- | --- | --- | --- | --- | --- |
|  |  |  | **HR (95%CI)** | ***P*-Value** | **HR (95%CI)** | ***P*-Value** | **HR (95%CI)** | ***P*-Value** |
| **All-cause death** |  |  |  |  |  |  |  |  |
| Power/mass>0.362 | 35 (14.8) | 4.7 (3.3-6.5) | 1.00 (ref) |  | 1.00 (ref) |  | 1.00 (ref) |  |
| Power/mass≤0.362 | 53 (22.3) | 7.3 (5.4-9.5) | 1.53 (1-2.35) | 0.051 | 1.30 (0.84-2.02) | 0.239 | 1.20 (0.77-1.86) | 0.431 |
| **Cardiovascular death** |  |  |  |  |  |  |  |  |
| Power/mass>0.362 | 24 (10.1) | 3.2 (2.1-4.8) | 1.00 (ref) |  | 1.00 (ref) |  | 1.00 (ref) |  |
| Power/mass≤0.362 | 33 (13.9) | 4.5 (3.1-6.4) | 1.38 (0.81-2.33) | 0.240 | 1.08 (0.61-1.9) | 0.790 | 1.06 (0.59-1.91) | 0.850 |
| **Noncardiovascular death** |  |  |  |  |  |  |  |  |
| Power/mass>0.362 | 11 (4.6) | 1.5 (0.7-2.6) | 1.00 (ref) |  | 1.00 (ref) |  | 1.00 (ref) |  |
| Power/mass≤0.362 | 20 (8.4) | 2.7 (1.7-4.2) | 1.78 (0.85-3.71) | 0.130 | 1.36 (0.66-2.8) | 0.400 | 1.62 (0.74-3.53) | 0.220 |
| **Hospitalization for heart failure** |  |  |  |  |  |  |  |  |
| Power/mass>0.362 | 32 (13.5) | 4.6 (3.2-6.5) | 1.00 (ref) |  | 1.00 (ref) |  | 1.00 (ref) |  |
| Power/mass≤0.362 | 55 (23.1) | 8.6 (6.5-11.2) | 1.84 (1.19-2.85) | 0.006 | 1.38 (0.88-2.17) | 0.155 | 1.74 (1.08-2.8) | 0.023 |
| **Primary endpoint** |  |  |  |  |  |  |  |  |
| Power/mass>0.362 | 51 (21.5) | 7.3 (5.5-9.7) | 1.00 (ref) |  | 1.00 (ref) |  | 1.00 (ref) |  |
| Power/mass≤0.362 | 76 (31.9) | 12.0 (9.4-15.0) | 1.62 (1.14-2.31) | 0.008 | 1.32 (0.92-1.91) | 0.133 | 1.58 (1.08-2.33) | 0.020 |

**Model 1：**adjusted for age, sex, race, region of enrolment (Americas versus Russia/Georgia) and randomization group.

**Model 2：**adjusted for model 1, additionally adjusted for NYHA functional class, stroke, atrial fibrillation and heart rate, creatinine and LVEF.

**Table S3. Risk of clinical outcomes by E/e’ ratio categories**

| **Outcomes** | **Event rate** | **Incidence rates, per 100 person-years** | **Unadjusted** | | **Model 1** | | **Model 2** |  |
| --- | --- | --- | --- | --- | --- | --- | --- | --- |
|  |  |  | **HR (95%CI)** | **P-Value** | **HR (95%CI)** | **P-Value** | **HR (95%CI)** | **P-Value** |
| **All-cause death** |  |  |  |  |  |  |  |  |
| E/e' ratio≤15 | 26 (10.5) | 3.2 (2.1-4.7) | 1.00 (ref) |  | 1.00 (ref) |  | 1.00 (ref) |  |
| E/e' ratio>15 | 62 (27.2) | 9.3 (7.1-11.9) | 2.98 (1.88-4.71) | <0.001 | 2.93 (1.85-4.64) | <0.001 | 2.73 (1.72-4.33) | <0.001 |
| **Cardiovascular death** |  |  |  |  |  |  |  |  |
| E/e' ratio≤15 | 19 (7.7) | 2.4 (1.4-3.7) | 1.00 (ref) |  | 1.00 (ref) |  | 1.00 (ref) |  |
| E/e' ratio>15 | 38 (16.7) | 5.7 (4.0-7.8) | 2.34 (1.35-4.03) | 0.002 | 2.38 (1.35-4.2) | 0.003 | 2.16 (1.22-3.82) | 0.008 |
| **Noncardiovascular death** |  |  |  |  |  |  |  |  |
| E/e' ratio≤15 | 7 (2.8) | 0.9 (0.3-1.8) | 1.00 (ref) |  | 1.00 (ref) |  | 1.00 (ref) |  |
| E/e' ratio>15 | 24 (10.5) | 3.6 (2.3-5.4) | 3.92 (1.68-9.15) | 0.002 | 3.68 (1.54-8.77) | 0.003 | 3.48 (1.45-8.35) | 0.005 |
| **Hospitalization for heart failure** |  |  |  |  |  |  |  |  |
| E/e' ratio≤15 | 30 (12.1) | 4.0 (2.7-5.7) | 1.00 (ref) |  | 1.00 (ref) |  | 1.00 (ref) |  |
| E/e' ratio>15 | 57 (25.0) | 9.9 (7.5-12.8) | 2.38 (1.53-3.71) | <0.001 | 1.96 (1.25-3.07) | 0.003 | 1.91 (1.22-2.99) | 0.005 |
| **Primary endpoint** |  |  |  |  |  |  |  |  |
| E/e' ratio≤15 | 46 (18.6) | 6.1 (4.5-8.2) | 1.00 (ref) |  | 1.00 (ref) |  | 1.00 (ref) |  |
| E/e' ratio>15 | 81 (35.5) | 14.0 (11.1-17.4) | 2.25 (1.57-3.24) | <0.001 | 2.05 (1.42-2.95) | <0.001 | 1.93 (1.33-2.79) | <0.001 |

**Model 1：**adjusted for age, sex, race, region of enrolment (Americas versus Russia/Georgia) and randomization group.

**Model 2：**adjusted for model 1, additionally adjusted for NYHA functional class, stroke, atrial fibrillation and heart rate, creatinine and LVEF.

**
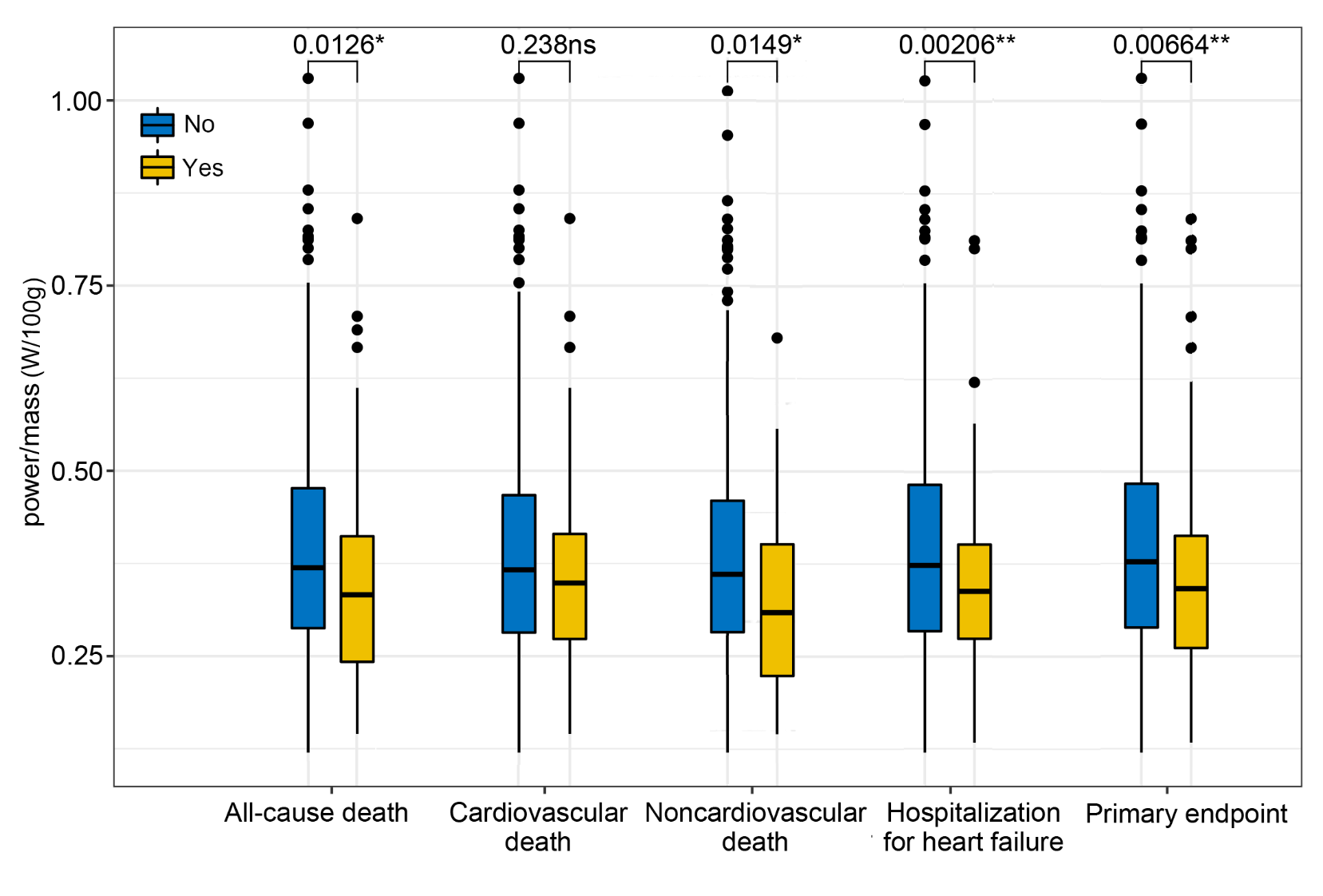
**

**Figure S1.** Difference of power/mass between HFpEF patients suffering from outcomes and those free from outcomes.

**
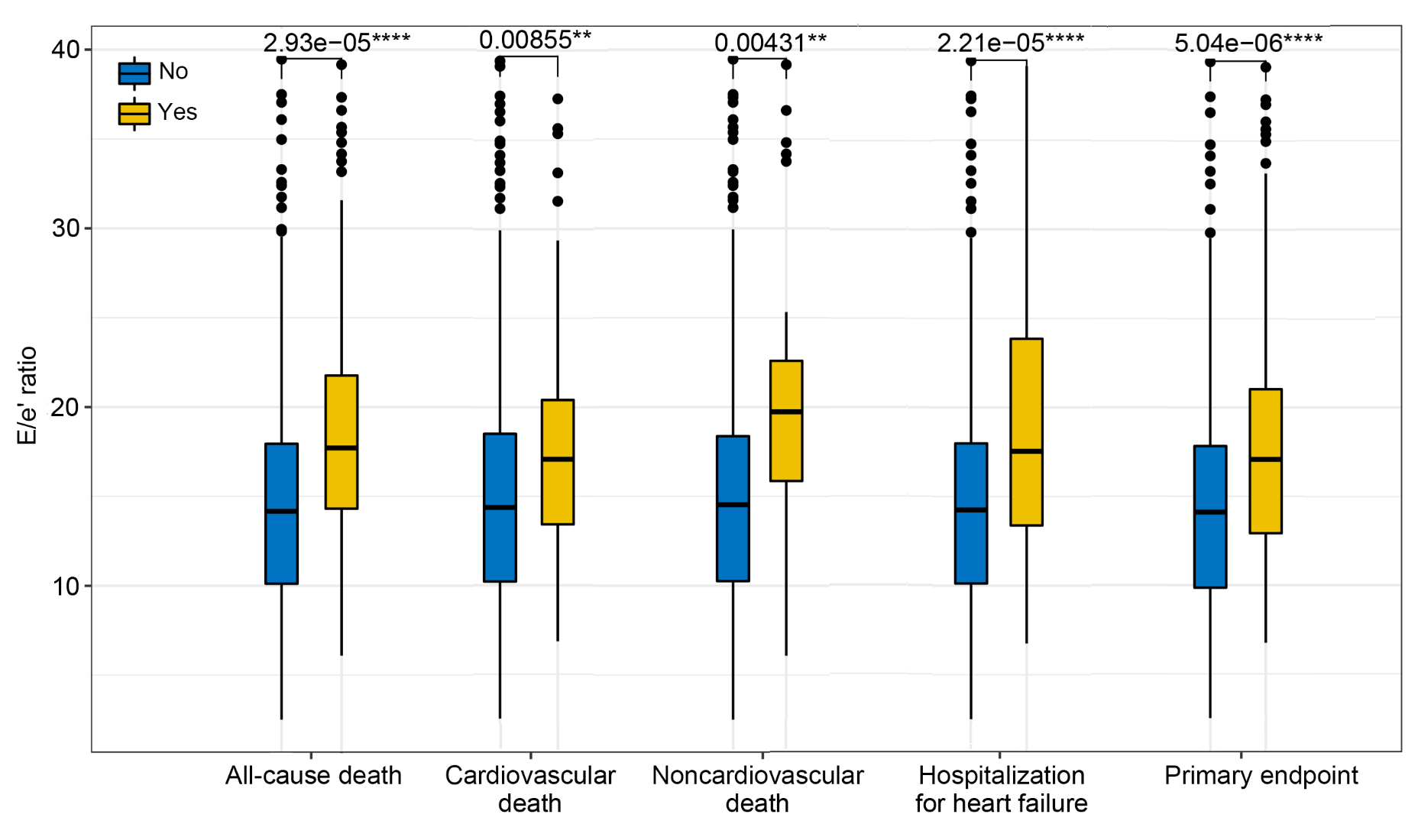
**

**Figure S2.** Difference of E/e’ ratio between HFpEF patients suffering from outcomes and those free from outcomes.
